# The Immunoregulatory Mechanisms of Human Cytomegalovirus from Primary Infection to Reactivation

**DOI:** 10.3390/pathogens14100998

**Published:** 2025-10-02

**Authors:** Xiaodan Liu, Chang Liu, Ting Zhang

**Affiliations:** 1 Laboratory of Hepatic AI Translation, Frontiers Science Center for Disease-Related Molecular Network, West China Hospital, Sichuan University, Chengdu 610041, China; xiaodanliu0822@gmail.com; 2 Interventional Diagnosis and Treatment Center, West China Hospital, Sichuan University, No. 37 Guoxue Alley, Chengdu 610041, China; 3 Department of General Surgery, West China Hospital, Sichuan University, Chengdu 610041, China; 4 Liver Transplant Center, Transplant Center, West China Hospital, Sichuan University, Chengdu 610041, China; 5 Room 1121, D5A Building, Chengdu Frontier Medical Science Center, No. 2222 Xinchuan Road, Chengdu 610213, China

**Keywords:** cytomegalovirus, immune evasion, reactivation

## Abstract

Human cytomegalovirus (HCMV) establishes lifelong latency following primary infection, residing within myeloid progenitor cells and monocytes. To achieve this, the virus employs multiple immune evasion strategies. It suppresses innate immune signaling by inhibiting Toll-like receptor and cGAS-STING pathways. In addition, the virus suppresses major histocompatibility complex (MHC)-dependent antigen presentation to evade T cell recognition. As the downregulation of MHC molecules may trigger NK cell activation, the virus compensates for this by expressing proteins such as UL40 and IL-10, which engage inhibitory NK cell receptors and block activating signals, thereby suppressing NK cell immune surveillance. Viral proteins like UL36 and UL37 block host cell apoptosis and necroptosis, allowing HCMV to persist undetected and avoid clearance. In settings of profound immunosuppression, such as after allogeneic hematopoietic stem cell transplantation (allo-HSCT) or solid organ transplantation, slow immune reconstitution creates a window for viral reactivation. Likewise, immunosenescence and chronic low-grade inflammation during aging increases the risk of reactivation. Once reactivated, HCMV triggers programmed cell death, releasing viral PAMPs (pathogen-associated molecular patterns) and host-derived DAMPs (damage-associated molecular patterns). This release fuels a potent inflammatory response, promoting further viral reactivation and exacerbating tissue damage, creating a vicious cycle. This cycle of inflammation and reactivation contributes to both transplant-related complications and the decline of antiviral immunity in the elderly. Therefore, understanding the immune regulatory mechanisms that govern the switch from latency to reactivation is critical, especially within the unique immune landscapes of transplantation and aging. Elucidating these pathways is essential for developing strategies to prevent and treat HCMV-related disease in these high-risk populations.

## 1. Introduction

Cytomegalovirus (CMV), a large double-stranded DNA virus, belongs to the herpesvirus family [1], establishes lifelong infection in humans and is highly prevalent worldwide. Studies show that CMV seroprevalence varies significantly across different countries and populations. In developing countries: in Sri Lanka, the seroprevalence was 94.9% in 2020 [2]; in Brazil, it was 88.5% in 2021 [3]; and in Romania, the seroprevalence among pregnant women was 93.7% during 2013–2016 and 95.0% during 2019–2022 [4]. In developed countries: in the United States and Canada, the seroprevalence among 18–19-year-olds was 49% and 25%, respectively, from 2005 to 2022, rising to 50–60% and 20–30% by the age of 38–39 [5]; in Spain, the seroprevalence was about 73.5% during 2010–2013 and 2020–2023 [6]; in Germany, it decreased from 63.7% in 1988–2018 to about 56.5% [7]; in Singapore, the seroprevalence among pregnant women was 71.7% in 2021 [8]; and in Israel, it was approximately 89% from 2013 to 2019 [9]. Overall, seroprevalence is generally higher in developing countries than in developed countries, and it gradually increases with age. In individuals with intact immune systems, CMV infection is typically asymptomatic. However, in immunocompromised individuals, CMV is prone to reactivation, often leading to a variety of serious clinical complications [10]. At the cellular level, CMV infection induces characteristic cytopathic effects. Although the virus is present in only 0.004–0.12% of peripheral blood monocytes, with each infected cell carrying 2 to 13 genome copies, the infection persists over time [11]. In the early stages of viral infection, cells become rounded. As the infection progresses, dense or granular inclusion bodies appear in both the nucleus and cytoplasm, accompanied by an increase in cell size. At the molecular level, the viral protein vMIA lowers intracellular ATP levels and inhibits mitochondrial phosphate transport, leading to impaired actin polymerization and cell rounding. Together, these alterations give rise to the characteristic cytopathic effects of CMV [12,13]. Considering the cellular and molecular characteristics of CMV infection, understanding its infection pathways, latency mechanisms, and reactivation triggers is crucial for developing effective strategies to prevent and treat CMV-associated diseases.

## 2. Primary Infection

### 2.1. Routes of Transmission and Susceptible Populations

Primary CMV infection commonly occurs during the perinatal period and infancy. If a woman is infected during pregnancy, approximately 32% of cases result in transplacental transmission to the fetus, leading to congenital infection [14]. In adults, CMV is mainly transmitted through sexual contact, and the virus spreads via infectious bodily fluids such as semen and genital secretions [15]. Individuals with weakened immune systems, including the elderly, patients with acquired immunodeficiency syndrome (AIDS), organ transplant recipients and those undergoing immunosuppressive therapy are at increased risk of primary CMV infection [16]. Furthermore, in these immunocompromised populations, not only is primary infection more likely, but the risk of CMV reactivation is also significantly higher. As such, regular screening, close monitoring, and preventive treatment strategies are particularly important in these groups.

### 2.2. Clinical Manifestations

In most cases, primary CMV infection in adults is asymptomatic; however, some individuals may develop a range of clinical manifestations, including arthritis, ulcerative colitis, hepatitis, and myocarditis. In addition, some patients may present with symptoms resembling mononucleosis characterized by persistent fever lasting 2–3 weeks, myalgia, and cervical lymphadenopathy [17]. In children, CMV infection can lead to complications such as hearing loss and cognitive impairment [18], although the vast majority of congenitally infected newborns (approximately 90%) show no clinical signs [19]. In immunocompromised patients, CMV infection may lead to neurological disorders, with the most common clinical manifestations including encephalitis, myelitis, and polyradiculopathy [20].

## 3. Latency

### 3.1. Types of Latently Infected Cells

All herpes viruses persist in the host by establishing latent infection in specific cells. Bone marrow-derived myeloid progenitor cells represent one of the key reservoirs for CMV latency [21]. The virus can establish a latent state in CD34^+^ progenitor cells (the precursors of B cells, T cells, and monocytes) [22], characterized by suppression of immediate early (IE) gene transcription, a restricted gene expression profile, and the absence of infectious virus production [23]. Another latent reservoir includes CD33^+^ myelomonocytic lineage granulocyte-macrophage progenitor cells (GM-Ps) [24]. In addition, CD14^+^ monocytes expressing B7-H4, as well as macrophages derived from them, have also been identified as sites of CMV latency and persistence [25]. The infected macrophages maintain the latency and long-term persistence of CMV by inhibiting apoptosis and limiting the cytopathic effects [26]. Macrophages are also susceptible targets and reservoirs for various viruses, such as influenza virus, chikungunya virus, human herpesviruses, and HIV, which can replicate effectively within them [27,28,29,30]. These findings suggest that CMV may preferentially target early-stage myeloid progenitors or influence pluripotent stem cells to differentiate into myeloid subsets more conducive to viral latency. CMV has also been found to establish latency in vascular endothelial cells, which form the inner lining of blood vessels [24], the phenomenon that may have implications for vascular health in the elderly.

The latent CMV genome can be found across a variety of organs, forming the molecular basis for human cytomegalovirus (HCMV) reactivation in multiple organ systems [31]. HCMV is among the most common infections following liver transplantation. Studies using the murine CMV (MCMV) model have identified liver sinusoidal endothelial cells (LSECs) as a potential site of viral latency in the liver [32]. However, latent CMV-infected endothelial cells have not been observed in the veins of seropositive individuals [33], suggesting that latency may exhibit tissue or species specificity and that murine models may not fully recapitulate the latency mechanisms of HCMV. Another mouse model found that kidney transplantation can induce activation of the major immediate early promoter (MIEP) of the virus [34], indicating that the kidney may also serve as a site of latency. Due to the complexity of HCMV latency and potential differences in latent viral reservoirs between HCMV and MCMV, the use of animal models has certain limitations. As a result, the precise identity of latently infected cell types within solid organs remains a matter of debate.

Because the currently known host cell types are highly conducive to CMV latency, no alternative cell types have been identified that are more suitable for supporting latent infection. During latency, viral gene expression is extremely low, and the number of latently infected cells is minimal, making detection difficult. In vitro models often fail to fully recapitulate the true latent state observed in vivo. Moreover, there is a lack of specific biomarkers to clearly distinguish latent infection from low-level viral activity, and no unified criteria exist for defining latency. Additionally, research interest in CMV may have shifted toward understanding regulatory pathways and immune evasion mechanisms, contributing to a slowdown in studies focused on latent reservoir cell types. Nonetheless, emerging technologies such as single-cell sequencing may offer new opportunities to advance this area of research.

### 3.2. Immune Evasion

CMV has evolved a highly effective latent infection strategy through millions of years of coevolution with its human host, making it challenging to eradicate. By sustaining low-level gene expression within host cells and employing multiple immune evasion tactics, CMV is able to remain latent over the long term, ensuring its survival and transmission [35]. These evasion mechanisms target various host defenses, including pattern recognition receptor (PRR)-mediated antiviral responses, MHC-mediated antigen presentation and intrinsic antiviral pathways such as apoptosis and autophagy.

#### 3.2.1. CMV Evasion of PRR-Mediated Antiviral Innate Immune Responses

**Figure 1:**
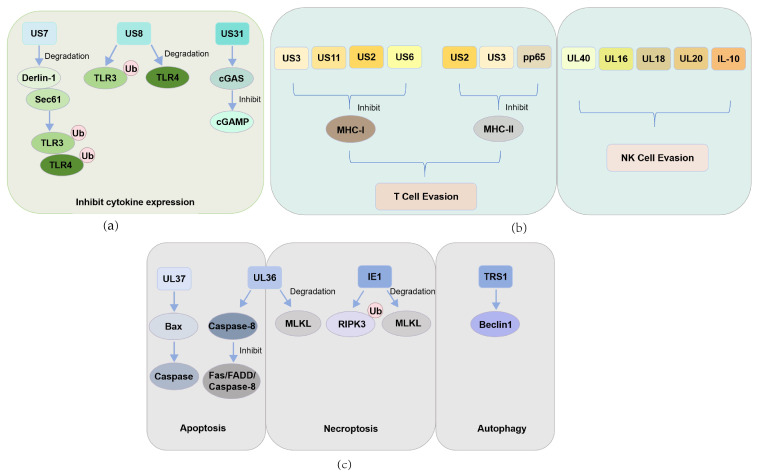
CMV encodes different proteins to evade host immune responses: (**a**) HCMV evades the host’s innate antiviral immune response mediated by pattern recognition receptors by encoding US7, US8, and UL31 proteins that promote the degradation of Toll-like receptors, inhibit the cGAS-STING signaling pathway, and subsequently suppress the expression of downstream antiviral cytokines; (**b**) HCMV employs multiple viral proteins to inhibit antigen presentation and evade immune clearance: US3, US11, US2, and US6 suppress MHC-I-mediated antigen presentation; US2, US3, and pp65 impair MHC-II presentation to escape T cell recognition; UL40, UL16, UL18, UL20, and viral IL-10 interfere with NK cell-mediated cytotoxicity; (**c**) HCMV interferes with host cell death pathways through multiple mechanisms to achieve immune evasion. The viral UL37 protein inhibits intrinsic apoptosis, while UL36 functions as a dual-action cell death suppressor that blocks both apoptosis and necroptosis. The IE1 protein suppresses necroptosis by inhibiting the functions of RIPK3 and MLKL. In addition, the autophagy-inhibiting protein TRS1 interacts with Beclin 1 to effectively block the autophagic process.

The innate immune system serves as the body’s first line of defense against pathogen invasion. When viruses infect host cells, intracellular PRRs detect viral nucleic acids and other pathogen-associated molecular patterns (PAMPs). This recognition triggers a cascade of intracellular signaling events and gene expression, leading to the production of inflammatory factors and cytokines. Ultimately, these processes activate the host’s natural immune response [36]. During CMV infection, viral proteins and nucleic acids are recognized as PAMPs by Toll-like receptors (TLRs) [37]. However, CMV has evolved strategies to evade these immune defenses by directly interfering with antiviral signaling pathways. For example, the HCMV-encoded membrane proteins US7 and US8 act as potent inhibitors of TLR3 and TLR4. US7, primarily localized in the endoplasmic reticulum (ER), has been shown to interact with ER-associated degradation components such as Derlin-1 and Sec61. In cells expressing US7, significant accumulation of ubiquitinated forms of TLR3 and TLR4 has been observed, suggesting that US7 may recruit E3 ubiquitin ligases to target these receptors for degradation via the ubiquitin-proteasome pathway. US8, which localizes to the Golgi apparatus or lysosomes, also interferes with TLR signaling. t disrupts the interaction between TLR3 and UNC93B1-an ER-resident protein critical for TLR function-leading to TLR3 ubiquitination and degradation. Additionally, US8 promotes the lysosomal targeting and degradation of TLR4. As a result, expression of US7 or US8 significantly suppresses TLR3- and TLR4-mediated transcription of genes such as IFN-β, CXCL10, and CXCL11 [38], effectively dampening the host’s innate immune response and facilitating viral infection or latency. Beyond TLRs, cytosolic DNA sensing is another crucial component of antiviral defense. The cyclic GMP-AMP synthase (cGAS) pathway is a major cytosolic surveillance mechanism that detects double-stranded DNA (dsDNA) from viruses. Upon binding dsDNA, cGAS generates the second messenger cyclic GMP-AMP (cGAMP), which activates the protein STING and triggers a signaling cascade that leads to the expression of type I interferons (IFN-I) and other inflammatory cytokines [39]. HCMV counters this pathway through the action of the viral protein UL31, which binds directly to cGAS, displacing viral DNA and preventing cGAMP synthesis. This interference blocks activation of STING and downstream expression of IFN-I and related antiviral effectors [40]. Through the targeted suppression of the cGAS-STING axis by UL31, HCMV evades immune surveillance and enhances its ability to persist within the host (Figure 1a).

Conventional immunomodulatory therapies have limited effectiveness in eliminating latent CMV infections. A deeper understanding of the virus’s immune evasion strategies may shed light on how CMV ensures its persistence and offer new directions for drug development. For instance, therapeutics that specifically target viral immune evasion proteins such as US7 and US8. These viral proteins employ distinct mechanisms to interfere with host immune responses, but whether they also act in coordination to suppress antiviral gene activation and collectively enhance the maintenance of latency remains an open question that warrants further investigation.

#### 3.2.2. CMV Evasion of T Cell- and NK Cell-Mediated Immune Surveillance

Although CMV can delay host defenses by suppressing innate immunity, the virus still faces the threat of adaptive immune responses, particularly T cell-mediated recognition and clearance. T cell responses are crucial for controlling CMV infection, as CMV-specific CD8^+^ T cells can recognize and kill viral proteins expressed at different stages of replication [41]. However, HCMV expresses multiple immune evasion proteins that block MHC-I and MHC-II-mediated antigen presentation, thereby weakening T cell responses. In the MHC-I pathway, US3 is synthesized during the IE phase of infection and retains fully assembled MHC-I complexes in the ER. US11, expressed at later stages, mediates degradation of MHC-I α chains through an ER-associated degradation (ERAD) pathway [42]. US2 can also bind specific lumenal domains of MHC-I heavy chains, triggering their retro-translocation from the ER into the proteasome and downregulating human leukocyte antigen (HLA)-A, HLA-G, and most HLA-B alleles [43]. US6 binds the transporter associated with antigen processing (TAP)/MHC-I complex and blocks TAP-mediated translocation of peptides from the cytosol into the ER [44]. Some proteins have dual functions; for example, US2 and US3 can also inhibit MHC-II signaling. In the MHC-II pathway, US2 recognizes and binds to HLA-DR α and HLA-DM α, triggering their degradation via the ERAD pathway [45]. US3 binds MHC-II α/β heterodimers in the ER and prevents association with the invariant chain (Ii), which is required for proper targeting to the acidic peptide-loading compartment (MIIC) [46]. The CMV protein pp65 misdirects HLA-DR molecules to lysosomes and degrades the α chain, blocking their surface transport and weakening CD4^+^ T cell responses [47]. Additionally, early in infection, CMV suppresses expression of CIITA, the MHC-II transcription factor, thereby inhibiting IFN-γ-induced MHC-II transcription; at later stages, the virus reduces Jak1 protein levels, impairing the Jak/STAT pathway. Blockade of STAT-1 phosphorylation further suppresses MHC-II expression [48].

While downregulation of MHC-I helps CMV evade CD8^+^ T cells, it can trigger natural killer (NK) cell “missing-self” responses. NK cells play a key role in immune surveillance by producing cytokines and directly killing target cells when they detect infection, tumors, or allogeneic tissue. Their distinctive feature is the ability to sense the absence of self MHC-I molecules, a process mainly mediated by inhibitory MHC-I receptors and NKG2 family receptors [49]. MHC downregulation during CMV infection could therefore activate NK cells. To counter this, CMV has evolved multiple mechanisms: the UL40-derived signal peptide stabilizes HLA-E and engages the inhibitory NKG2A receptor on NK cells, mimicking “self” signals [50]; UL16 blocks NKG2D binding to its ligands, UL16-binding protein (ULBP) and major histocompatibility complex class I chain-related protein B (MICB), suppressing NK cell activation [51]; US18 and US20 cooperate to degrade MICA via lysosomes, further reducing NKG2D-mediated activation [52]. Additionally, CMV-encoded IL-10 decreases classical MHC-I/II expression while upregulating the non-classical HLA-G allele, helping the virus escape NK cell recognition [53] (Figure 1b).

However, the host immune system is not a passive bystander. Research has shown that activating NK cell receptors exhibit significant peptide dependence, serving as important weapons against viral evasion. In particular, during HCMV infection, adaptive NKG2C^+^ NK cells can accurately discriminate UL40-derived peptides presented by HLA-E, with even a single amino acid difference affecting their recognition and activation. Different UL40 peptides determine the extent of NKG2C^+^ NK cell expansion and, in conjunction with inflammatory factors, promote their differentiation and long-term persistence, thereby generating NK cell populations with memory-like characteristics within the host [54]. Furthermore, HLA-E-binding peptides derived from HCMV pp65 can also induce the expansion and antiviral activity of NKG2C-dependent adaptive NK cells [55]. Although NKG2C binds HLA-E with lower affinity than inhibitory receptors, its high dependence on specific viral peptides enables it to play a critical role during HCMV infection and reactivation, shaping highly variable NK cell repertoires among individuals [56]. These findings indicate that while CMV weakens NK cell activation through multiple mechanisms, the host counteracts via peptide-dependent activating pathways, resulting in a continuous dynamic interplay that ultimately shapes the complexity of the immune response.

In summary, CMV not only blocks MHC-dependent antigen presentation to evade T cell detection but also modulates non-classical HLA molecules and inhibits NKG2 receptor pathways to avoid NK cell surveillance. These layered strategies collectively ensure CMV’s long-term persistence and reactivation.

#### 3.2.3. CMV Evades Apoptosis, Necroptosis, and Autophagy

When PRRs recognize PAMPs and T cells are activated via antigen presentation by MHC molecules, host cells undergo a stress response, eliciting a range of stress-induced processes such as autophagy, apoptosis, and necroptosis [37]. These programmed cell deaths are essential not only for eliminating the virus and restricting the spread of infection, but also for modulating innate immune responses. Consequently, by interfering with PRR signaling pathways, viruses can suppress the production of inflammatory factors and potentially influence cell death, thereby evading host immunity at multiple levels.

#### 3.2.4. CMV Inhibits Apoptosis

Apoptosis can be divided into intrinsic and extrinsic pathways. In vertebrates, the primary intrinsic apoptotic pathway relies on increased mitochondrial outer membrane permeability mediated by two pro-apoptotic proteins of the BCL2 family, BAX and BAK. This leads to the release of cytochrome c and other mitochondrial factors, which subsequently trigger the activation of caspase-9, followed by the downstream activation of effector proteins such as caspase-3, caspase-6, and caspase-7 [57]. CMV encodes several anti-apoptotic inhibitors, such as the viral mitochondria-localized inhibitor of apoptosis (vMIA) encoded by the UL37 gene of HCMV [58]. vMIA binds directly to BAX, preventing its role in increasing mitochondrial membrane permeability and blocking the release of cytochrome c, thereby inhibiting the activation of downstream caspases and ultimately suppressing intrinsic apoptosis [59]. vMIA contains two essential structural domains: one for mitochondrial localization and another for direct interaction with BAX [60]. This structural organization underlies its ability to inhibit apoptosis and highlights the close relationship between viral protein structure and function. In mice, MCMV encodes the m38.5 protein, which exerts a similar anti-apoptotic function by interacting with mouse BAX and recruiting it to the mitochondria [61]. Although vMIA and m38.5 share low sequence homology, they perform analogous functions, suggesting that viruses can preserve essential functions while altering protein sequences to evade immune recognition. Suppressing intrinsic apoptosis is critical for viral survival during the early stages of infection, as it helps prevent premature host cell death, thereby creating a favorable environment for viral replication and latency.

Extrinsic apoptosis is initiated by extracellular signals, typically triggered by the binding of ligands to tumor necrosis factor receptors (TNFRs). Upon activation, TNFRs recruit Fas-associated death domain protein (FADD), leading to the formation of the Fas/FADD/Caspase-8 death-inducing signaling complex, which in turn initiates the apoptotic cascade [62]. The HCMV-encoded protein viral inhibitor of caspase-8-induced apoptosis (vICA), produced by the UL36 gene, binds to the precursor form of caspase-8, preventing its activation and blocking its recruitment to the Fas/FADD/Caspase-8 complex, thereby exerting an anti-apoptotic effect [58]. MCMV M36, a homolog of HCMV UL36 [63], functions similarly to a caspase-8 inhibitor. By interrupting the transmission of extrinsic apoptotic signals, M36 suppresses apoptosis and supports the establishment of viral latency. Through precise interference with the extrinsic apoptosis pathway via UL36, HCMV demonstrates a highly adapted strategy for evading host immune surveillance. The conservation of this mechanism across species further highlights its potential value as a therapeutic target in antiviral intervention.

#### 3.2.5. CMV Suppression of Necroptosis

Although apoptosis is the most common form of cell death, studies have shown the existence of multiple caspase-independent death pathways, such as necroptosis, which can occur independently of apoptotic signaling [64]. Receptor-interacting protein kinase 1 (RIPK1) was the first factor identified as being involved in this process [64]. Through its C-terminal RIP homotypic interaction motif (RHIM), RIPK1 can form homo- or heteromeric complexes with receptor-interacting protein kinase 3 (RIPK3), leading to the activation of RIPK3 [65]. RIPK3 is an essential kinase in the necroptosis pathway [66]. It activates the downstream effector mixed lineage kinase domain-like (MLKL) via phosphorylation through its N-terminal kinase domain [67], thereby triggering necroptosis. Studies have found that MLKL expression is significantly reduced in fibroblasts 48 h after HCMV infection. This is due to the viral protein pUL36, which interacts directly with MLKL and promotes its degradation [68], effectively blocking necroptosis and preventing premature death of the infected cell. Beyond mediating cell death, MLKL also regulates multiple cellular processes, including magnesium-dependent ER homeostasis, endosomal trafficking, extracellular vesicle formation, autophagosome formation, and autophagy [69]. Therefore, pUL36-mediated degradation of MLKL may not only suppress cell death but also influence the viral replication cycle, including processes such as virion assembly, intracellular transport, and cell-to-cell spread, potentially by promoting vesicle formation to enhance viral dissemination among host cells. As previously noted, pUL36 can inhibit both caspase-8–dependent apoptosis [58] and MLKL-mediated necroptosis, making it a dual-function inhibitor of programmed cell death and a potentially valuable therapeutic target in HCMV infection. However, the precise mechanisms by which pUL36 mediates MLKL degradation, as well as the details of its interaction with both MLKL and caspase-8, remain to be fully elucidated. Additionally, whether MLKL degradation directly affects viral replication, assembly, or spread requires further investigation. Nevertheless, these findings provide a theoretical foundation and new direction for the development of antiviral therapies targeting molecular interactions such as pUL36-MLKL.

The initiation of HCMV replication depends on the transcription of viral IE genes [70], among which IE1 plays a pivotal role. Studies have shown that IE1 can interact with RIPK3 in a RHIM-independent manner, thereby interfering with RIPK3-mediated necroptotic signaling. This interference may be associated with the ubiquitination of RIPK3, although the specific type of ubiquitin modification involved remains unclear. Expression of MLKL is known to be regulated by IFNs [71], particularly IFN-β, which significantly upregulates MLKL levels. However, in the presence of IE1, both MLKL mRNA and protein levels are reduced by approximately 50% [72], suggesting that IE1 inhibits necroptosis through a dual mechanism, directly disrupting RIPK3 function and indirectly suppressing MLKL expression. In addition to RIPK1 and RIPK3, the Z-DNA binding protein 1 (ZBP1), which also contains a RHIM domain, can interact with RIPK3 to recruit and activate its kinase activity [73], leading to MLKL phosphorylation and the initiation of necroptosis [74]. In murine models, the N-terminal RHIM domain of the MCMV-encoded M45 protein can mimic the interaction between RIPK1 or ZBP1 and RIPK3 [75], competitively blocking RIPK3 activation and effectively suppressing necroptosis. The evolution of viral proteins with multi-target inhibitory functions and RHIM-based competitive strategies highlights the significant threat these host defense pathways pose to viral survival. ZBP1, as a PRR, can detect abnormal nucleic acid structures—such as Z-form DNA or RNA—generated during viral infection, and directly initiate necroptotic signaling. This dual role in both sensing and execution underscores its importance in the host’s innate immune defense. Several DNA viruses, including HCMV, MCMV, and HSV, have been shown to activate ZBP1, suggesting that Z-form nucleic acids may be produced during infection. However, whether these structures arise from the viral genome, replication intermediates, or are stress-induced byproducts of the host cell remains an open question.

#### 3.2.6. CMV Interference with the Autophagy Pathway

In addition to inhibiting apoptosis and necroptosis, CMV can also disrupt the host’s autophagic response. Studies have shown that in human primary fibroblasts infected with HCMV, autophagy is almost completely suppressed within 24 h. Beclin 1, a key regulator in autophagosome formation, plays a central role in initiating autophagy. The HCMV-encoded protein TRS1, known for its anti-autophagic function, can interact with Beclin 1 and block the initiation of the autophagic process [76]. Compared to apoptosis and necroptosis, autophagy is considered a more “gentle” mechanism of cellular clearance. By binding to Beclin 1, TRS1 effectively prevents the assembly of the autophagy initiation complex, thereby protecting the virus from degradation via autophagy. Although some studies have uncovered aspects of how CMV modulates autophagy, many questions remain unanswered regarding the interaction between the virus and the host autophagy system, and further investigation is needed (Figure 1c).

CMV regulates host cell death pathways through a range of viral proteins, targeting key nodes within apoptotic and necroptotic signaling to suppress host immune responses and facilitate viral latency. By inhibiting cell death, CMV limits the activation of the immune system, thereby maintaining a latent state. These immune evasion strategies not only help the virus avoid clearance but may also create a more favorable environment to enhance its replication. Latent infection is one of the key mechanisms by which CMV persists long-term within host cells (Table 1). Therefore, accurately detecting viral presence during latency and effectively preventing reactivation during immunosuppressive therapy are crucial for disease management. Immunotherapeutic approaches—such as immune boosters or checkpoint inhibitors—show promise in enhancing the host’s ability to eliminate latent virus. However, given CMV’s sophisticated immune evasion tactics and diverse reservoir sites, relying solely on immune-boosting strategies may prove insufficient for complete viral clearance. A more targeted, multifaceted treatment approach remains necessary.

## 4. Reactivation

Following primary infection, HCMV establishes lifelong latency but can reactivate when myeloid progenitors differentiate or under stress signals (e.g., hypoxia, inflammation) [77]. In immunocompetent hosts, cell-mediated immunity usually controls reactivation. However, in immunocompromised individuals or transplant recipients on immunosuppressive therapy, HCMV reactivation can become uncontrolled, causing severe, even life-threatening, complications.

### 4.1. Mechanisms and Clinical Manifestations of HCMV Reactivation After Transplantation

Allogeneic hematopoietic stem cell transplantation (allo-HSCT) remains the only curative option for various hematologic malignancies and severe disorders [78]. However, reactivation of HCMV after transplantation is one of the most common opportunistic infections. It may result either from a primary infection or from the reactivation of latent virus, posing a significant challenge to patient prognosis.

Multiple factors contribute to the risk of HCMV reactivation. Among them, the HCMV serostatus of both donor and recipient plays a critical role. Studies have shown that approximately 60–70% of HCMV-seropositive recipients experience viral reactivation following allo-HSCT. Even when the donor is seropositive and the recipient is seronegative, reactivation can still occur in about 10% of cases [79,80]. The transplantation process itself, along with the use of immunosuppressive agents such as corticosteroids and cyclosporine [81], often triggers a systemic inflammatory response, leading to elevated levels of cytokines like TNF and IL-2 [82,83]. While these cytokines may exhibit antiviral properties under certain conditions, in the context of latent HCMV infection, they can paradoxically promote viral reactivation. Mechanistically, proinflammatory cytokines such as TNF-α activate transcription factors like NF-κB and AP-1. These factors form p65/p50 complexes, which translocate into the nucleus and bind to the immediate early (IE) promoter of HCMV, thereby initiating viral replication [84,85]. Moreover, in studies of sepsis-induced immunosuppression, up to 80% of patients were found to experience HCMV reactivation. These individuals exhibited reduced T cell counts, significantly higher expression of PD-1 on both CD4^+^ and CD8^+^ T cells, and elevated levels of IL-6 and IL-10 [86]. HCMV further disrupts immune homeostasis by promoting T cell exhaustion, enhancing cytokine production, and sustaining a state of chronic inflammation.

Following allo-HSCT, HCMV reactivation not only exacerbates immune dysregulation but is also closely associated with a range of clinical complications. For instance, primary HCMV infection has been shown to significantly increase the risk of hemorrhagic cystitis (HC) [87]. When HCMV reactivation occurs in combination with bacterial bloodstream infections, HC tends to progress more rapidly, presents with more severe symptoms, and is linked to poorer outcomes [88]. HCMV reactivation may also have complex effects on immune reconstitution. Some studies suggest that it may be involved in the relapse of acute myeloid leukemia (AML) [89]. Conversely, other research has indicated an association between HCMV reactivation and a reduced risk of early relapse in AML patients [90]. However, data from the Center for International Blood and Marrow Transplant Research (CIBMTR) showed no significant link between HCMV reactivation and AML relapse [91]. As such, the role of HCMV reactivation in tumor recurrence remains controversial and warrants further in-depth investigation.

In solid organ transplantation, HCMV infection remains one of the leading pathogens causing post-operative complications. Among lung transplant recipients, HCMV pneumonia is strongly associated with secondary viremia and increased mortality [92]. As a frequent tissue-invasive disease following lung transplantation, HCMV pneumonia presents with non-specific clinical manifestations, typically including fever, dyspnea, non-productive cough, and deteriorating pulmonary function [93]. Studies have shown that transplant patients who remain free of HCMV pneumonia during the first six months post-operation demonstrate significantly higher 5-year survival rates compared to those who developed HCMV pneumonia and received antiviral therapy [94], underscoring the critical importance of early infection prevention for long-term outcomes. For liver transplant recipients, HCMV infection and reactivation most commonly manifest clinically as fever, leukopenia, and thrombocytopenia [95]. Hepatic artery thrombosis is also more frequently observed in patients with HCMV infection [96]. This serious vascular complication can lead to interruption of graft blood flow and biliary necrosis, ultimately resulting in graft failure or even death if not promptly identified and managed.

**Figure 2:**
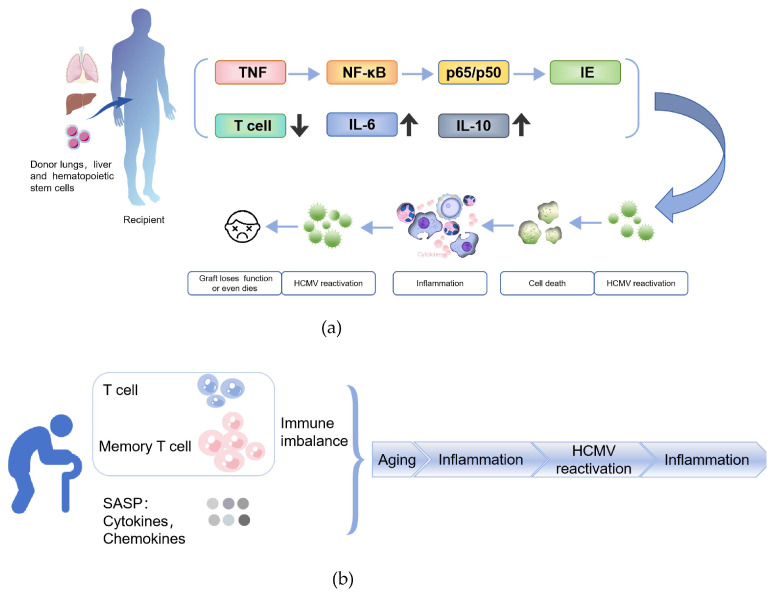
Mechanisms of HCMV reactivation in immunocompromised individuals: (**a**) After transplantation, high levels of cytokines can activate transcription factors and promote viral replication, thereby forming a cycle of viral reactivation—cell death—continued enhancement of inflammatory response—more viral activation; (**b**) Immunosenescence leads to reduced generation of naïve T cells and abnormal expansion of memory T cells, disrupting immune homeostasis. Meanwhile, senescent cells secrete large amounts of proinflammatory factors, forming the SASP, which ultimately establishes a self-reinforcing cycle: diminished immune function promotes inflammatory activation, which triggers CMV reactivation, leading to further immune decline.

Immunosuppressive therapy is widely used in organ transplantation and the treatment of immune-mediated diseases to suppress the host immune response and prevent graft rejection. However, these drugs can also impair the host’s immune surveillance against viruses such as HCMV, thereby increasing the risk of latent viral reactivation. Although CMV has acquired multiple immune evasion strategies throughout its long co-evolution with the host—such as encoding viral proteins that inhibit apoptosis and necroptosis—it cannot entirely escape host defenses, particularly within the complex inflammatory environment that follows transplantation. As a result, infected cells may still undergo necrotic death. Cell death is essential for maintaining tissue homeostasis, with approximately 10^11^ senescent, dysfunctional, or infected cells being cleared daily [97]. Upon ubiquitination, RIPK1 participates in assembling the TNFR1 complex, stabilizing the inhibitor of kappa B kinase (IKK) complex, degrading IκBα, and activating NF-κB, which promotes the release of proinflammatory cytokines, potentially contributing to latent viral reactivation. Conversely, when deubiquitinated, RIPK1 can mediate FADD/caspase-8-dependent apoptosis or activate RIPK3 to phosphorylate MLKL and induce necroptosis [98]. In addition, ZBP1, as another key regulatory factor, can also recruit RIPK3 via its RHIM domain, promoting MLKL activation and membrane permeabilization, thereby triggering necroptosis. Necroptosis of infected cells releases viral PAMPs and host DAMPs, which further trigger programmed cell death pathways and inflammatory responses. The reactivation of HCMV establishes a positive feedback loop-viral reactivation induces partial necroptosis and chronic inflammation, which then drives further reactivation (Figure 2a). Progressive loss of immune cells, epithelial cells, and other host cells weakens immune function, resulting in T-cell exhaustion, structural tissue damage, and potential graft failure. This demonstrates that CMV reactivation is not simply viral replication but a complex process involving cell death, inflammation, and immune dysregulation.

Despite considerable advances in understanding CMV reactivation, important questions remain unresolved. In particular, the mechanisms involved in identifying early biomarkers and the detailed regulatory networks that control virus–host interactions are not yet fully understood. Improved early detection capabilities and personalized treatment approaches will be crucial for reducing CMV-related complications and improving patient outcomes.

### 4.2. HCMV Reactivation Driven by Immunosenescence

Aging is an unavoidable physiological process in all living organisms, with the associated immunosenescence resulting in compromised immune responses. Consequently, the host becomes more susceptible to infections and shows reduced ability to respond to new threats [99], thereby exacerbating age-related diseases such as cardiovascular disorders and neurodegenerative conditions.

A hallmark of immunosenescence is the decline in T cell function, reduced regenerative capacity of the immune system, and the establishment of a chronic inflammatory state. Thymic atrophy and diminished hematopoietic function in the bone marrow restrict the production of new T cells [100], leading to a gradual loss of immune surveillance over new pathogens and latent viruses. As a vital component of the immune system, the bone marrow undergoes structural and functional changes during aging, leading to a reduced number of immune progenitor cells. This impairs the production of lymphocytes and monocytes, further accelerating the overall decline in immune function and particularly weakening the response to early viral infections. In this context, HCMV reactivation triggers a robust immune response, leading to the abnormal accumulation of HCMV-specific memory T cells-particularly CD28^−^ effector memory T cells in the elderly [101]. These cells exhibit reduced T cell receptor (TCR) diversity and functional exhaustion [102], gradually occupying substantial immunological space and contributing to “memory inflation” [103]. This phenomenon further suppresses responses to novel antigens, exacerbates immune exhaustion, and weakens the host’s capacity to control HCMV reactivation. In addition, senescence-associated mitochondrial dysfunction (SAMD) also impairs antiviral responses in aging. SAMD induces excessive production of reactive oxygen species (ROS), activates DNA damage response (DDR) and the NLRP3 inflammasome pathway, driving the release of proinflammatory cytokines such as IL-1β and IL-18. It also activates the NF-κB signaling pathway, thereby enhancing the transcription of proinflammatory mediators. These pathways synergistically constitute the core mechanism by which SAMD drives the senescence-associated secretory phenotype (SASP) [104,105]. This persistent inflammatory state not only impairs immune defense mechanisms but may also contribute to the development of various age-related diseases, including metabolic disorders, atherosclerosis, and cancer (Figure 2b). CMV reactivation has been increasingly observed in SARS-CoV-2-infected patients. One of the key immunological features of COVID-19 is profound immune dysregulation, particularly characterized by T cell dysfunction and exhaustion [106], which significantly impairs the host’s immune surveillance of latent herpesviruses and facilitates viral reactivation. In addition, the proinflammatory response induced by SARS-CoV-2 may further contribute to the reactivation of latent viruses. Notably, in patients with post-acute sequelae of SARS-CoV-2 infection (PASC), elevated levels of proinflammatory cytokines such as IL-1β, IL-6, and TNF may create a microenvironment that favors viral reactivation [107]. These cytokines can promote the transition of latent viruses into active replication by modulating viral gene expression. In addition, many patients with severe COVID-19 need strong immunosuppressive treatments, including high-dose corticosteroids and IL-6 antagonists [108], which further compromise antiviral immunity and may increase the risk of CMV reactivation. Under these conditions, the reactivation of herpesviruses such as CMV, Epstein–Barr virus (EBV) may elicit or exacerbate clinical manifestations resembling those of PASC, including persistent fatigue, cognitive dysfunction, and dyspnea [109], thereby further impeding patient recovery.

Immunosenescence is commonly associated with chronic inflammation, which viral infections can worsen by stimulating proinflammatory cytokine release. This sustained inflammatory environment accelerates immune deterioration, establishing a self-reinforcing cycle: diminished immune function promotes inflammatory activation, which triggers CMV reactivation, leading to further immune decline. During the SARS-CoV-2 pandemic, this cycle has become particularly dangerous for elderly and immunocompromised patients, making CMV reactivation an increasingly complex clinical challenge.

## 5. Conclusions

CMV infects nearly all humans and, through millions of years of co-evolution with its host, has developed highly sophisticated mechanisms for latency and immune evasion. This prolonged coexistence not only reflects CMV’s remarkable adaptation to the host immune system but also reveals the diversity and complexity of immune recognition and responses following infection. In immunocompetent individuals, most can harbor CMV for extended periods without obvious symptoms, and its latent infection therefore commonly accompanies the human aging process, being regarded as a prevalent feature of “normative aging.” Latent infection can alter the composition of the immune system, but in most cases, these changes are compatible with health and may only contribute to immune aging under specific circumstances [110].

CMV reactivation is more than a virological event; it is a critical clinical challenge that requires close attention. Reactivation can involve multiple organ systems—including the liver, gastrointestinal tract, and lungs—and is particularly severe in immunocompromised individuals, often resulting in serious clinical consequences. Immunosuppressive therapies, proinflammatory cytokines, and specific immune markers interact to drive this pathogenic process. Further investigation into the systemic interactions of CMV will be essential for elucidating its complex disease mechanisms.

CMV employs sophisticated strategies to establish persistent infection by simultaneously exploiting immune evasion mechanisms and disrupting critical signaling pathways. The virus exhibits remarkable selectivity in targeting specific cell death pathways, especially those triggered by cytokine signaling or viral PAMPs. Through this multilayered interference, CMV systematically dismantles host defenses, ultimately reshaping the immune microenvironment to favor viral replication and dissemination. Deciphering these complex virus–host interactions at the molecular level remains essential for understanding CMV pathogenesis and disease progression.

From a clinical perspective, the development of personalized antiviral strategies should be prioritized. Approaches such as real-time viral load monitoring and immune biomarker profiling can enable precise management of high-risk patients, thereby reducing CMV-related morbidity and mortality and improving overall outcomes. This precision medicine approach is particularly promising for addressing complications associated with aging and immune dysfunction.

## Figures and Tables

**Table 1:** Evasion of host antiviral responses by CMV-encoded proteins.

Antiviral Pathway	Virus Type	CMV-Encoded Regulatory Proteins	Target Host Proteins	Reference
PRR recognition	HCMV	US7	TLR3, TLR4	[38]
		US8	TLR3, TLR4	[38]
		UL31	cGAS	[40]
T cell	HCMV	US3	MHC-I, MHC-II	[42,46]
		US11	MHC-I	[42]
		US2	MHC-I, MHC-II	[43,45]
		US6	MHC-I	[44]
		pp65	MHC-II	[47]
NK cell	HCMV	UL40	HLA-E	[50]
		UL16	NKG2D	[51]
		UL18	MICA	[52]
		UL20	MICA	[52]
		IL-10	HLA-G	[53]
Apoptosis	HCMV	UL36	Caspase-8	[58]
		UL37	BAX	[59]
	MCMV	M36	Caspase-8	[63]
		M38.5	BAX	[61]
Necroptosis	HCMV	UL36	MLKL	[68]
		IE1	RIPK3	[70]
		IE1	MLKL	[72]
	MCMV	M45	RIPK3	[75]
Autophagy	HCMV	TRS1	Beclin1	[76]

## Abbreviations

The following abbreviations are used in this manuscript:

HCMV	Human cytomegalovirus
MHC	Major histocompatibility complex
Allo-HSCT	Allogeneic hematopoietic stem cell transplantation
PAMPs	Pathogen-associated molecular patterns
DAMPs	Damage-associated molecular patterns
CMV	Cytomegalovirus
IgG	Immunoglobulin G
AIDS	Acquired immunodeficiency syndrome
IE	Immediate early
GM-Ps	Granulocyte-macrophage progenitor cells
MCMV	Murine cytomegalovirus
LSECs	Liver sinusoidal endothelial cells
MIEP	Major immediate early promoter
PRR	Pattern recognition receptor
TLRs	Toll-like receptors
ER	Endoplasmic reticulum
cGAS	Cyclic GMP-AMP synthase
dsDNA	Double-stranded DNA
cGAMP	Cyclic GMP-AMP
IFN-I	Type I interferons
ERAD	ER-associated degradation
HLA	Human leukocyte antigen
TAP	Transporter associated with antigen processing
Ii	Invariant chain
MIIC	MHC class II compartment
NK	Natural killer
ULBP	UL16-binding protein
MICB	Major histocompatibility complex class I chain-related protein B
vMIA	Viral mitochondria-localized inhibitor of apoptosis
TNFRs	Tumor necrosis factor receptors
FADD	Fas-associated death domain protein
vICA	Viral inhibitor of caspase-8-induced apoptosis
RIPK1	Receptor-interacting protein kinase 1
RHIM	RIP homotypic interaction motif
RIPK3	Receptor-interacting protein kinase 3
MLKL	Mixed lineage kinase domain-like
ZBP1	Z-DNA binding protein 1
HC	Hemorrhagic cystitis
AML	Acute myeloid leukemia
CIBMTR	Center for International Blood and Marrow Transplant Research
IKK	Inhibitor of kappa B kinase
TCR	T cell receptor
SAMD	Senescence-associated mitochondrial dysfunction
ROS	Reactive oxygen species
DDR	DNA damage response
SASP	Senescence-associated secretory phenotype
PASC	Post-acute sequelae of SARS-CoV-2 infection
EBV	Epstein–Barr virus
